# Hematological Parameters as an Early Marker of Deep Vein Thrombosis in Diabetes Mellitus: An Observational Study

**DOI:** 10.7759/cureus.36813

**Published:** 2023-03-28

**Authors:** Shyam V, Satyapriya Mohanty, Debasish Das, Amit Ghosh, Rituparna Maiti, Pranati Nanda

**Affiliations:** 1 Physiology, All India Institute of Medical Sciences, Bhubaneswar, Bhubaneswar, IND; 2 Cardiothoracic Vascular Surgery, All India Institute of Medical Sciences, Bhubaneswar, Bhubaneswar, IND; 3 Cardiology, All India Institute of Medical Sciences, Bhubaneswar, Bhubaneswar, IND; 4 Pharmacology, All India Institute of Medical Sciences, Bhubaneswar, Bhubaneswar, IND

**Keywords:** neutrophil-to-lymphocyte ratio, platelet-to-lymphocyte ratio, diabetes mellitus, total leukocyte count, deep vein thrombosis

## Abstract

Background

Although the association between the presence of diabetes mellitus and the development of deep vein thrombosis (DVT) is well known, the role of novel biomarkers in predicting the development of DVT in diabetic patients is not yet known to a large extent. Studies have shown that complete blood count (CBC) and CBC-derived parameters such as neutrophil-to-lymphocyte ratio (NLR) and platelet-to-lymphocyte ratio (PLR) can be used as surrogate markers to detect DVT. This study was conducted to assess the utility of NLR and PLR as a marker of DVT in diabetic patients.

Methodology

This case-control study was conducted among a calculated sample size of 109 DVT patients in one arm and 109 non-DVT patients in another arm. Hematological tests including total leucocyte count, differential leucocyte count, total neutrophil count, total lymphocyte count, total platelet count, NLR, and PLR were performed.

Results

We found a significant difference in NLR and PLR between the DVT and the non-DVT groups. In addition, we found that NLR and PLR were significantly higher in the diabetic group, indicating the presence of inflammation in association with diabetes mellitus. Analysis of the receiver operating characteristic curve showed that at a cut-off value of 2.83, NLR can detect DVT in diabetic patients with 67% sensitivity and 92% specificity. Similarly, PLR at a cut-off value of 131.46 can detect DVT in diabetic patients with 56% sensitivity and 90% specificity.

Conclusions

We conclude that NLR and PLR are novel inflammatory markers that can help in the early detection of DVT in diabetic patients.

## Introduction

White blood cells are associated with acute and chronic inflammation. Neutrophilto-lymphocyte ratio (NLR) is an easily available marker for the evaluation of chronic inflammatory disorders [1]. It is derived from the total neutrophil and total lymphocyte counts of the complete blood count (CBC). NLR has been shown to have clinical relevance in metabolic syndrome [2], atherosclerosis, and cardiovascular disease [3]. The prevalence of diabetes mellitus in India is around 7.3% [4], and 14.9% of patients with diabetes mellitus develop deep vein thrombosis (DVT) during their lifetime [5]. DVT carries a grave prognosis in diabetes mellitus with one-year mortality of around 4.7-15.4% [6]. Post-thrombotic syndrome develops in 25-50% of patients with DVT within two years which seriously affects their quality of life [7].

DVT involves the activation of a network of coagulation factors, and the process is hastened by inflammatory mediators within the microenvironment. It is associated with impaired fibrinolysis [8]. Systemic inflammation has a modulatory role in thrombosis [9]. Hence, the CBC may provide an idea about the inflammatory status of the patient, which can indirectly indicate the procoagulant/prothrombotic state. From the CBC, various clues regarding the inflammatory status of the body can be obtained. CBC parameters that can provide a wide range of details regarding the type and duration of the inflammation are available even at the grassroots levels of the healthcare system. They can be widely used and easily interpreted to detect various inflammatory conditions. The derived parameters such as NLR and platelet-to-lymphocyte ratio (PLR) are recently being used as new diagnostic criteria for various inflammatory disorders. Few studies have shown the association of these derived parameters with acute DVT [5]. DVT has been found to be associated with high monocyte counts [8]. In addition, these parameters have been shown to be associated with the vascular complications of diabetes mellitus [9,10]. To date, very few studies have been conducted among diabetes patients, particularly in association with vascular complications such as DVT, using the CBC and its derived parameters as a marker of inflammation. There are still ongoing controversies regarding the use of CBC-derived parameters as an early diagnostic marker of DVT or to assess the prognosis. Although some recent studies have shown an association of CBC parameters with DVT, it has not been utilized as a diagnostic marker. Similarly, recent studies have shown the association of CBC parameters with complicated diabetes mellitus [11], but they fail to answer the question of whether the increase in the inflammatory parameters is a result of the thrombotic microvascular complications or due to the diabetic status per se. Hence, there might be a correlation between the CBC-derived parameters and the diabetes status of an individual [12]. The use of CBC-derived parameters as an early predictor of DVT in diabetic patients is an area that has not been investigated and needs to be studied further. As 14.9% of diabetic patients may develop DVT, it is a burden on the healthcare facility which needs to be addressed. Hence, the objective of our study was to determine a suitable early marker for DVT in diabetic patients and assess the utility of NLR and PLR as a marker of DVT to reduce the associated mortality and morbidity with DVT in these patients.

This study aimed to investigate different hematological parameters in patients with DVT with and without diabetes mellitus. The primary objective was to evaluate the mean difference in NLR and PLR between patients with DVT and healthy controls. The secondary objective was to evaluate the difference in NLR and PLR in patients with DVT with and without diabetes mellitus.

## Materials and methods

This case-control study evaluated the changes in the total leukocyte count, NLR, and PLR in DVT patients attending the Department of Cardiovascular Surgery, All India Institute of Medical Sciences (AIIMS), Bhubaneswar, India. In addition, we compared the abovementioned parameters with age and gender-matched controls from AIIMS, Bhubaneswar, India. The sample size was calculated using a sample size calculation formula for case-control studies (https://communitymedicine4asses.files.wordpress.com/2021/10/sample-size-formula-for-case-control-studies) with a power of 80% and an attrition rate of 15%. After obtaining approval from the Institutional Ethical Committee of AIIMS, Bhubaneswar (approval number: IEC/AIIMS BBSR/PG Thesis/2019-20/10), the study was conducted with 109 participants in each group between September 15, 2019, to December 15, 2020. Patients with hematologic diseases, active infections, malignancies, and cardiac, renal, or hepatic disorders were excluded from the study. Those who refused to give consent were also excluded from the study. The study initially enrolled 250 patients, 20 of whom were not willing to give consent for participation, and another 12 patients were excluded based on the exclusion criteria. Finally, 238 patients were enrolled in this study. They were divided into the following two groups: group A (DVT) and group B (non-DVT). Group A or the DVT arm consisted of 119 patients, and group B or the non-DVT arm consisted of 119 patients. Group A or the DVT arm was again divided into the following two subgroups: subgroup 1 with diabetes mellitus (n = 39), and subgroup 2 without diabetes mellitus (n = 70). Group B or the non-DVT arm was divided into the following two subgroups: subgroup 1 with diabetes mellitus (n = 52), and subgroup 2 without diabetes mellitus (n = 57). All participants were interviewed using a self-made questionnaire. Their demographic data were collected, their medical history was assessed, and their medical reports were reviewed. Patients with fasting blood sugar levels of more than 126 mg/dL, postprandial blood sugar levels of more than 200 mg/dL, and HbA1c of more than 6.5% were grouped as diabetic [13,14]. Patients’ weight (in kg), height (in m), heart rate (in beats per minute), and blood pressure (in mmHg) were recorded in the outpatient department for future reference. All participants underwent blood sample collection by a finger prick method, and capillary blood was obtained. Total leukocyte counts were determined manually using Turk’s dilution fluid in Neubauer’s chamber. Total platelet counts were determined manually in Neubauer’s chamber using an ammonium oxalate solution. Differential leukocyte counts were determined in a peripheral blood smear obtained by a finger prick and stained with Leishman stain. Using the percentage values obtained from the differential count and the total leukocyte counts, total neutrophil count, and total lymphocyte count were calculated as total neutrophil counts = % of neutrophils in differential count × total leukocyte count/100 cells/mm^3^, and total lymphocyte count = % of lymphocytes in differential count × total leukocyte count/100 cells/mm^3^. From these values, NLR and PLR were calculated.

The data were analyzed using R software (2022). The continuous variables were expressed as mean ± SD, and the categorical variables were expressed as median and interquartile range. All continuous data were tested for normality using the Shapiro-Wilk test. Normally distributed data in both the groups were tested by unpaired Student’s t-test, and non-normally distributed data were tested using the Mann-Whitney test to evaluate the difference between the groups. Spearman’s correlation analysis was performed to correlate two continuous variables. Receiver operator characteristic (ROC) curve analysis was performed to determine a cut-off value of NLR and PLR for detecting DVT. Sensitivity, specificity, and area under the curve (AUC) for delineation of cut-off points for NLR and PLR were also calculated.

## Results

Table 1 presents the baseline variables of DVT and non-DVT groups. The median age, height, and weight of DVT group participants were not significantly different from that of the non-DVT group, but the median body mass index (BMI) was significantly higher in the DVT group. A higher proportion of DVT patients had a history of varicose veins compared to the non-DVT group.

**Table 1:** Baseline variables of study participants. BMI: body mass index; SBP: systolic blood pressure; DBP: diastolic blood pressure; MI: myocardial infarction; DVT: deep vein thrombosis; DM: diabetes mellitus

Variables	DVT (n = 109)	Non-DVT (n = 109)	P-value
Age (years)	47 (37–64)	47 (35–62)	0.466
Male	52.3%	51.4%	0.8922
Height (m)	1.62 (1.54–1.68)	1.63 (1.54–1.7)	0.078
Weight (kg)	68.00 (62–72)	69.00 (59–75)	0.852
BMI (kg/m^2^)	26.09 (24.7–27.5)	24.91 (23.8–26.22)	0.001
SBP (mmHg)	134.00 (126–142)	128.00 (122–136)	<0.0001
DBP (mmHg)	84.00 (80–90)	80.00 (76–84)	<0.0001
Smokers (%)	32.1%	28.4%	0.5554
Alcoholic (%)	42.2%	43.1%	0.8911
Varicose vein patients (%)	10.1%	1.8%	0.01005
History of stroke/MI (%)	4.6%	2.8%	0.4712
Family history of DVT (%)	13.8%	2.8%	0.003147
Family history of DM (%)	23.9%	16.5%	0.177

Table 2 presents the comparison of variables among diabetic and non-diabetic patients having DVT. Diabetic patients having DVT were elderly, had higher total leucocyte count, total neutrophil count, total lymphocyte count, higher platelet count, higher monocyte count, NLR, PLR, and higher HbA1C (p < 0.05) than diabetic patients without DVT. Table 3 presents the comparison of variables among diabetic and non-diabetic patients without DVT. Diabetic patients without DVT had higher BMI, higher total leucocyte count, total neutrophil count, total lymphocyte count, platelet count, monocyte count, NLR, PLR, and higher HbA1C (p < 0.05) than non-diabetic patients without DVT.

**Table 2:** Comparison of variables among DVT patients with and without diabetes mellitus BMI: body mass index; NLR: neutrophil-to-lymphocyte ratio; PLR: platelet-to-lymphocyte ratio; HbA1C: hemoglobin A1C; SBP: systolic blood pressure; DBP: diastolic blood pressure; DVT: deep vein thrombosis; DM: diabetes mellitus

Variables	DVT group having DM (n = 39)	DVT group without DM (n = 70)	P-value
Age (years)	55 (44–62)	44 (35–54.75)	0.005
Height (m)	1.60 (1.52–1.67)	1.63 (1.54–1.68)	0.420
Weight (kg)	68.00 (59–72)	69.50 (62.2–73.5)	0.258
BMI in (kg/m^2^)	25.99 ± 2.64	26.14 ± 2.16	0.744
Total leukocyte count	12,087.18 ± 2,245.02	9,927.00 ± 2,799.652	0.0001
Total neutrophil count	8,828.46 ± 1,610.37	6,593.86 ± 1,937.66	0.0001
Total lymphocyte count	2,820 (2,235–3,665)	2,945 (2,417–3,725)	0.633
Platelet count	407,000 (359,500–439000)	352,500 (284,500–398,000)	0.003
Monocyte count	150 (109–210)	115 (100–147.5)	0.008
NLR	3.04 (2.56–3.71)	2.18 (1.86–2.5)	0.0001
PLR	137.73 (19.9–164.76)	114.49 (87.53–144.2)	0.038
HbA1C (%)	6.90 (6.4–8.2)	5.4 (5–5.6)	0.0001
SBP (mmHg)	138.00 (126–146)	134 (126–142)	0.432
DBP (mmHg)	82.00 (80–90)	85 (80–90)	0.668
Total cholesterol (mg/dL)	190 (170–213)	190 (180–200)	0.742
Smokers (%)	28.2%	34.3%	0.51
Alcoholics (%)	33.3%	47.1%	0.1617

**Table 3:** Comparison of variables among non-DVT patients with and without DM. BMI: body mass index; NLR: neutrophil-to-lymphocyte ratio; PLR: platelet-to-lymphocyte ratio; HbA1C: hemoglobin A1C; SBP: systolic blood pressure; DBP: diastolic blood pressure; DVT: deep vein thrombosis; DM: diabetes mellitus

Variables	Non-DVT group having DM (n = 52)	Non-DVT group without DM (n = 57)	P-value
Age (years)	46.5 (42–61)	48 (37–61)	0.400
Weight (kg)	65 (58–76)	69 (62–74)	0.729
BMI (kg/m^2^)	26.04 ± 2.92	22.22 ± 1.8	0.0001
Total leukocyte Count	9,404.42 ± 1,719.949	6,559.65 ± 1,179.45	0.0001
Total neutrophil count	6,175.77 ± 1,271.60	3,725.61 ± 982.88	0.0001
Total lymphocyte count	3,066.04 ± 956.56	2,734.74 ± 580.65	0.029
Monocyte count	80 (50–100)	40.00 (30–80)	0.0001
Platelet count	299,473.08 ± 74,252.36	202,259.65 ± 65,224.66	0.0001
NLR	2.13 (1.71–2.64)	1.84 (1.38–2.08)	0.001
PLR	95.88 (83.1–116.8)	69.45 (56.59–91.17)	0.0001
HbA1C (%)	7.15 (6.5–8.6)	5.4 (5.2–5.5)	0.000
SBP (mmHg)	130 (120–140)	126 (122–132)	0.010
DBP (mmHg)	80 (76–84)	80 (76–84)	0.677
Total cholesterol (mg/dL)	192.15 ± 23.654	183.91 ± 17.211	0.039
Smokers (%)	32.7%	24.6%	0.3473
Alcoholics (%)	40.4%	45.6%	0.5819

**Figure 1:**
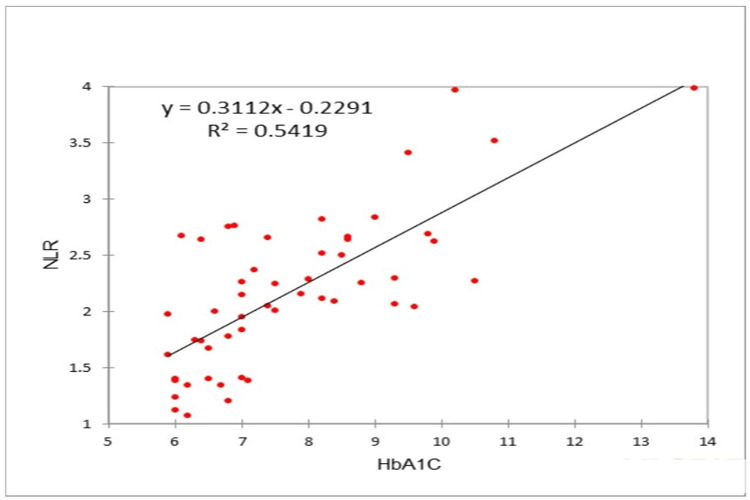
Correlation between NLR and HbA1C (significant at p-value <0.05). NLR: neutrophil-to-lymphocyte ratio; HbA1C: hemoglobin A1C

**Figure 2:**
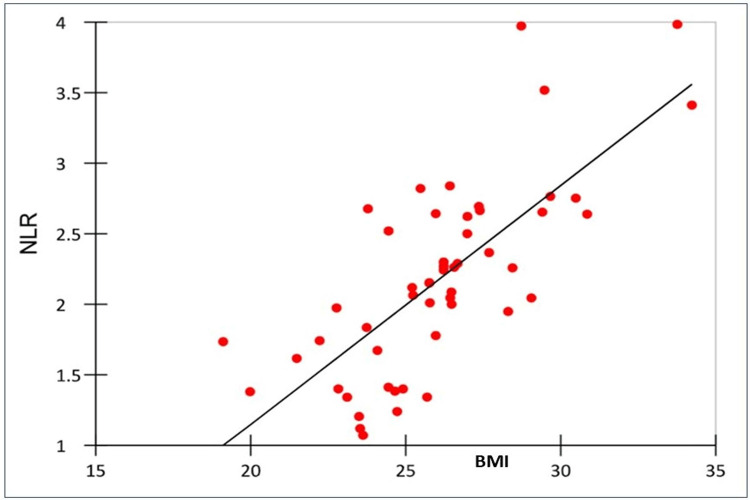
Correlation between NLR and BMI. NLR: neutrophil-to-lymphocyte ratio; BMI: body mass index

The correlation between NLR and HbA1C was analyzed and the result revealed a positive correlation (r = 0.7361), and it was statistically significant (p = 0.001) (Figure 1). The correlation between NLR and BMI has been analyzed and the result revealed a positive correlation between the two (r = 0.7337), and it was statistically significant (p < 0.001) (Figure 2).

**Figure 3:**
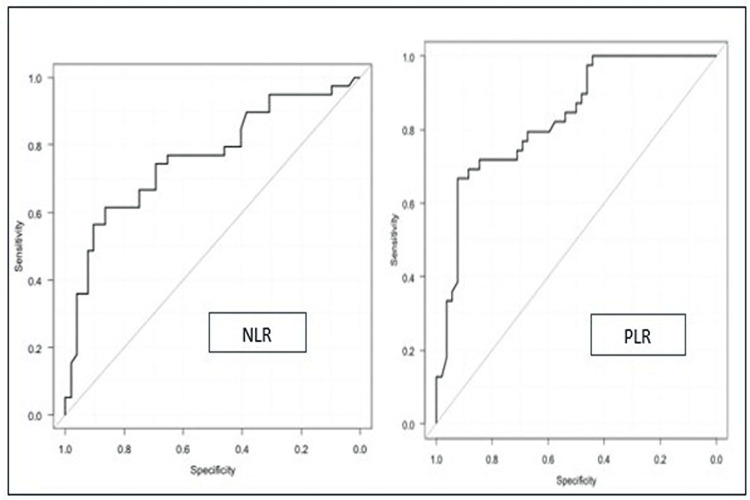
ROC curve for NLR and PLR in DVT. ROC: receiver operating characteristic; NLR: neutrophil-to-lymphocyte ratio; PLR: platelet-to-lymphocyte ratio; DVT: deep vein thrombosis

**Figure 4:**
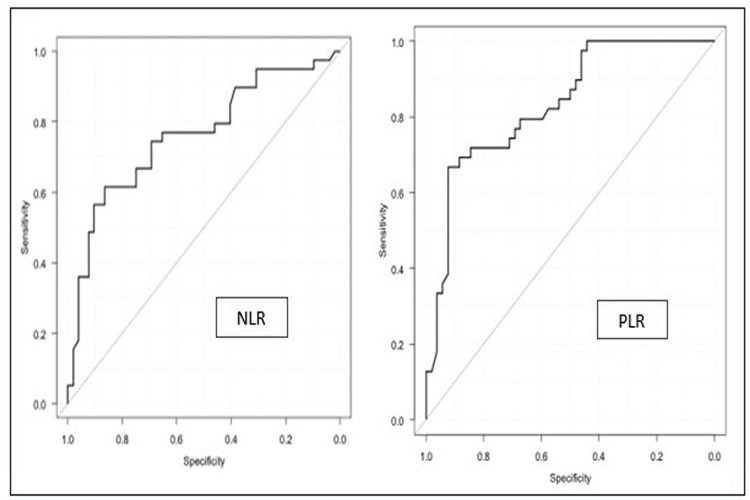
ROC of NLR and PLR for DVT in diabetic patients. ROC: receiver operating characteristic; NLR: neutrophil-to-lymphocyte ratio; PLR: platelet-to-lymphocyte ratio; DVT: deep vein thrombosis

The cut-off value for NLR with ROC analysis (AUC = 0.722, confidence interval (CI) = 65.6-78.9) to differentiate DVT from non-DVT was 2.164 (Figure 3) with 63% (53.5-72.3%) sensitivity and 65% (55.4-74%) specificity for diagnosing DVT. NLR value of 2.164 had a positive predictive value of 64.5% and a negative predictive value of 64%. The cut-off value for PLR with ROC analysis (AUC = 0.746, CI = 68.2-81.1) to differentiate DVT from non-DVT was 102.71 (Figure 3) with 68% (58.3-76%) sensitivity and 67% (57.3-76%) specificity. PLR value of 102.71 had a positive predictive value of 67.3% and a negative predictive value of 67.6%. The cut-off value for NLR with ROC analysis (AUC = 0.833, CI = 0.75-0.91) to differentiate DVT from non-DVT among diabetic patients was 2.83 (Figure 4) with 67% (49.8-81%) sensitivity and 92% (79-97%) specificity. NLR value of 2.83 had a positive predictive value of 83.9% and a negative predictive value of 78.3%. The cut-off value for PLR with ROC analysis (AUC = 0.762, CI = 0.66-0.86) to differentiate DVT from non-DVT among diabetic patients was 131.46 (Figure 4) with 56% (39.6-72.2%) sensitivity and 90% (79-97%) specificity. PLR value of 131.46 had a positive predictive value of 81.5% and a negative predictive value of 73.4%.

## Discussion

The NLR value in the DVT group was 2.37, which was significantly higher than the NLR value of 1.98 in the control group. Elevated NLR is a better marker of enhanced inflammation in DVT patients because NLR takes into account the neutrophil count and the lymphocyte count, with neutrophils serving as the first line of defense and lymphocytes regulating inflammation. PLR was found to be significantly higher in the DVT group compared to the control group. PLR acts as a combined marker of platelet aggregation and inflammatory status as it takes into account two components, namely, platelets and lymphocytes. Our findings were similar to those reported by Ming et al. [5], Erden et al. [15], and Tural et al. [16]. Taking NLR as a parameter for ROC analysis, the AUC for NLR was 0.722, which indicates that an increase in NLR serves as a predictive marker of DVT. The sensitivity and specificity of NLR to detect DVT were found to be 63% and 65%, respectively, at a cut-off value of 2.164. Similar results were reported by Erden et al. with an AUC of 0.739, a sensitivity of 72%, and a specificity of 67% at an NLR cut-off value of 2.12. Other studies by Farah et al. [17], Mouabbi et al. [18], Rinaldi et al. [19], Tural et al. [16], and Sujana et al. [20] also reported similar results. On ROC analysis of PLR, we found out that the AUC for PLR was 0.746. At a cut-off value of 102.71, the PLR positivity predicted DVT with 68% sensitivity and 67% specificity. Similar trends were found in the studies of Ming et al. [4] and Erden et al. [15]. Using the above-mentioned cut-off values, we assessed the utility of the double positivity of NLR and PLR in predicting DVT, and it was found that the double positivity of both NLR and PLR was able to predict DVT with a sensitivity of 63% (53.5-72.3%) and a specificity of 67%. We also correlated NLR and PLR values with HbA1C to determine any correlation between the inflammatory status and long-standing glycemic control in diabetic patients. In our study, the NLR and PLR positively correlated with the levels of HbA1C, which is a marker of long-term blood sugar control. The positive association between NLR, as well as PLR with HbA1C, can be attributed to the increased levels of inflammation associated with uncontrolled blood glucose levels. Our study established that the novel markers NLR and PLR can predict DVT in diabetic individuals beyond values of 2.83 and 131.46, respectively. These simple novel biomarkers can predict DVT in diabetic patients with reliable sensitivity and specificity even in small peripheral hospitals.

## Conclusions

NLR and PLR are novel biomarkers to detect DVT even in diabetic patients. Our study demonstrated that NLR and PLR were significantly (p < 0.05) higher in patients with DVT compared to patients without DVT. NLR and PLR were significantly higher among diabetic patients with DVT compared to non-diabetic patients with DVT. NLR as a diagnostic parameter has a sensitivity of 67% and specificity of 92% in detecting DVT among diabetic patients, and PLR as a diagnostic parameter has a sensitivity of 56% and specificity of 90% in detecting DVT among diabetic patients. These two novel biomarkers will add to the prediction and prevention of DVT among diabetic patients in the near future.
